# Differential regulation of Janus kinase 3 (JAK3) in bovine preovulatory follicles and identification of JAK3 interacting proteins in granulosa cells

**DOI:** 10.1186/s13048-016-0280-5

**Published:** 2016-10-28

**Authors:** Kalidou Ndiaye, Amélie Castonguay, Gabriel Benoit, David W. Silversides, Jacques G. Lussier

**Affiliations:** 1 Département de biomédecine vétérinaire, Faculté de médecine vétérinaire, Centre de recherche en reproduction animale (CRRA), Université de Montréal, P.O. Box 5000, St-Hyacinthe, Québec J2S 7C6 Canada; 2 Faculté de médecine vétérinaire, Département de biomédecine vétérinaire, Université de Montréal, 3200 Rue Sicotte, St-Hyacinthe, Québec J2S 2M2 Canada

**Keywords:** Janus kinase (JAK), Ovary, Granulosa cells, Yeast two-hybrid, STAT proteins

## Abstract

**Background:**

Janus kinase 3 (JAK3) is a member of the membrane-associated non-receptor tyrosine kinase protein family and is considered predominantly expressed in hematopoietic cells. We previously identified *JAK3* as a differentially expressed gene in granulosa cells (GC) of bovine preovulatory follicles. The present study aimed to further investigate JAK3 regulation, to identify protein binding partners and better understand its mode of action in bovine reproductive cells.

**Results:**

GC were obtained from small follicles (SF), dominant follicles at day 5 of the estrous cycle (DF), and ovulatory follicles, 24 h following hCG injection (OF). RT-PCR analyses showed greatest expression of *JAK3* in GC of DF, while *JAK3* expression was downregulated in OF (*P* < 0.0001). In addition, there was a 5- and 20-fold reduction of *JAK3* steady-state mRNA levels in follicular walls, respectively at 12 and 24 hours post-hCG as compared to 0 h (*P* < 0.05). Similarly, *JAK3* expression was downregulated by the endogenous LH surge. These results were confirmed in western blot analysis showing weakest JAK3 protein amounts in OF as compared to DF. Yeast two-hybrid screening of a DF-cDNA library resulted in the identification of JAK3 partners in GC that were confirmed by co-immunoprecipitation and included leptin receptor overlapping transcript-like 1 (LEPROTL1), inhibin beta A (INHBA) and cyclin-dependent kinase inhibitor 1B (CDKN1B). In functional studies using bovine endometrial cells, JAK3 increased phosphorylation of STAT3 and cell viability, while the addition of JANEX-1 inhibited JAK3 actions.

**Conclusion:**

These results support a physiologically relevant role of JAK3 in follicular development and provide insights into the mode of action and function of JAK3 in reproductive tissues.

**Electronic supplementary material:**

The online version of this article (doi:10.1186/s13048-016-0280-5) contains supplementary material, which is available to authorized users.

## Background

Janus kinase 3 (JAK3) belongs to a family of membrane-associated intracellular non-receptor tyrosine kinase proteins that mediate signals initiated by cytokine and growth factor receptors through the JAK/STAT pathway [1, 2]. Other members of the Janus family include JAK1, JAK2 and tyrosine kinase 2 (TYK2). In contrast to the ubiquitous expression of JAK1, JAK2 and TYK2 [3, 4], JAK3 is predominantly expressed in immune cells and is involved in signal transduction by interleukin (IL) receptors that share the common gamma chain (γc) of the type I cytokine receptor family [5–8]. Non-receptor tyrosine kinases are involved in diverse cellular processes including proliferation, differentiation, cell migration and survival [9–13]. In various systems, JAK signal transduction occurs via a well-characterized JAK/STAT pathway. JAKs activate this pathway by binding to cytokine receptors leading to the recruitment of STAT proteins, which are then phosphorylated. After undergoing JAK-mediated phosphorylation, the STAT transcription factors dimerize and translocate to the nucleus where they regulate the transcription of specific genes [14, 15].

In mammals, the JAK/STAT pathway is the principal signaling mechanism for a wide array of cytokines and growth factors. Different JAKs and STATs are recruited based on the specific tissue and the receptors engaged in the signaling event [16]. Although the canonical JAK/STAT pathway is simple and direct, the pathway components can regulate or be regulated by members of other signaling pathways, including those involving the ERK MAP kinase, phosphatidylinositide 3-kinases (PI3K), and receptor tyrosine kinase (RTK)/Ras/MAPK pathways [17]. Effector proteins that contribute to JAK/STAT events include the signal-transducing adapter molecules (STAMs), which facilitate the transcriptional activation of specific target genes, and the stat-interacting proteins (StIP) that can associate with JAKs and unphosphorylated STATs to facilitate JAK/STAT pathway [18–20]. Conversely, the suppressors of cytokine signaling (SOCS), the protein inhibitors of activated stats (PIAS) and the protein tyrosine phosphatases (PTP) are major negative regulators of the JAK/STAT pathway [21–23]. These data indicate that various proteins outside the basic pathway machinery could influence the JAK/STAT signaling.

JAK3 encodes for a 125-kDa protein made of seven JAK homology (JH) domains. The carboxyl JH1 region of JAK3 contains the activation loop, a region that includes the tyrosine kinase catalytic domain [15]. The kinase activity of JAK proteins depends on their phosphorylation at tyrosine residues in the activation loop of the kinase domain. Multiple sites of autophosphorylation have indeed been identified in this enzymatically active JH1 domain [24–26]. The JH2 domain contains the catalytically inactive pseudo-kinase domain, which is tandemly linked to the N site of the JH1 domain and represents a unique feature of JAK proteins in contrast to other tyrosine kinases. Despite the lack of catalytic activity, the pseudo-kinase domain is required for suppression of basal activity of tyrosine kinases and for cytokine-inducible activation of signal transduction [27]. The amino terminus region of JAK3 is composed of a SH2-like domain (JH3 and JH4 domains) and a 4.1, ezrin, radixin, moesin (FERM) homology domain (JH6 and JH7 domains). The FERM domain interacts with the kinase domain, positively regulates the catalytic activity and is critical for receptor binding, signal transduction and maintenance of kinase integrity [28].

*JAK3* is primarily expressed in hematopoietic cells and the JAK/STAT pathway has been widely investigated in immune cells, but JAK3 has also been found in a wide range of tissues of both hematopoietic and non-hematopoietic origin [29]. We previously identified *JAK3* as a differentially expressed gene in granulosa cells of bovine dominant follicles using a gene expression profiling approach [30]. *JAK3* was identified among a list of other genes that were down-regulated in granulosa cells of bovine ovulatory follicles following human chorionic gonadotropin (hCG) injection as compared to growing dominant preovulatory follicles during the estrous cycle. It is well documented that the cyclic ovarian activity results in profound modifications that require spatio-temporal coordination of proliferation, apoptosis and differentiation of various cell types within the follicle leading to changes in gene expression. Of interest, granulosa cells play a critical role in these reproductive functions as they contribute to steroid hormone synthesis [31], oocyte maturation [32], and corpus luteum formation after ovulation [33]. Many factors such as follicle-stimulating hormone receptor (FSHr) in small and growing follicles, luteinizing hormone receptor (LHr) in ovulatory follicles, steroid hormones (estradiol and progesterone) and growth factors are produced by GC and affect follicular growth, ovulation and differentiation into a functional corpus luteum. Consequently, the regulation of granulosa cell proliferation and function is complex and depends on the precise regulation and activation of specific target genes. This regulation is essential for normal follicular development and timely production of paracrine factors as it affects the physiological state of the dominant preovulatory follicle. For instance, the transcription of specific genes that control the growth of a bovine dominant preovulatory follicle is rapidly downregulated or silenced in granulosa cells as a result of LH-mediated increases in intracellular signaling [30]. These observations demonstrate the critical importance of gene regulation studies during the final stages of follicular development as well as their interactions and mode of action. In this regard, we identified JAK3 as a candidate gene associated with follicular growth and dominance. We report JAK3 differential regulation and binding partners in bovine granulosa cells as well as its effects in cell proliferation.

## Results

### JAK3 expression is differentially regulated during follicular development

**Fig. 1:**
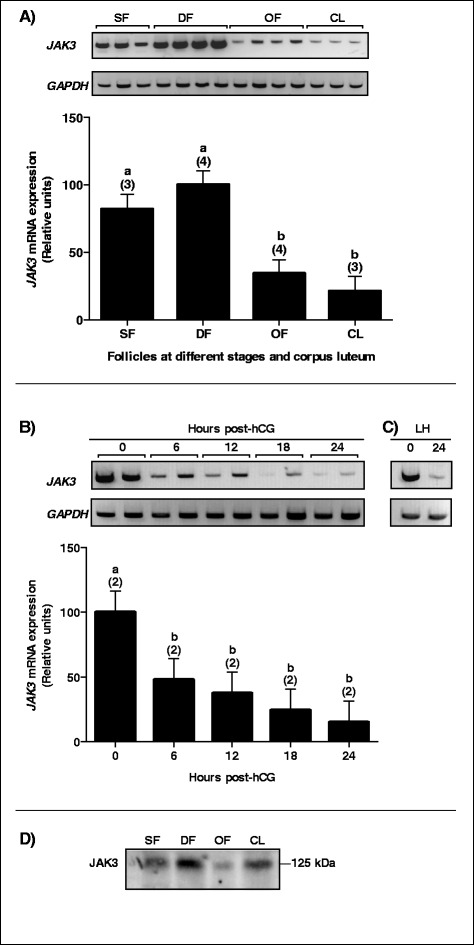
*JAK3* mRNA expression and regulation in bovine follicles. Total RNA extracts of GC from small follicles (SF), dominant follicles (DF), ovulatory follicles (OF), and corpus luteum at day 5 (CL) were analyzed by RT-PCR for *JAK3* with *GAPDH* used as reference. **a** Gel analysis of *JAK3* in different groups of follicles and CL and corresponding histograms. *JAK3* mRNA expression was strongest in DF and was significantly decreased in OF and CL (*P* < 0.0001). **b** Gel analysis of *JAK3* mRNA regulation in hCG-induced follicular walls (FW) isolated from OF at 0, 6, 12, 18 and 24 h (hrs) after hCG injection and corresponding histograms. *JAK3* mRNA was markedly decreased in FW 6 h post-hCG compared to its expression before hCG treatment (*P* < 0.01) and reached its weakest expression 24 h post-hCG as compared to 0 h (*P* < 0.0001). **c** Gel analysis of *JAK3* mRNA regulation by endogenous LH. Similar to hCG, a decrease in *JAK3* expression was observed 24 h after endogenous LH (LH) surge as compared to 0 h. **d** Representative JAK3 protein expression and regulation in bovine follicles. Total protein extracts of GC from SF, DF, OF, and CL were analyzed by western blot using anti-JAK3 antibodies. The strongest JAK3 protein expression was observed in the DF while weakest expression was observed in OF reflecting the regulation of the mRNA. Data are presented as least-square means ± SEM, and the number of independent samples per group is indicated in parenthesis

Expression of *JAK3* is significantly reduced in ovulatory follicles (OF) following hCG injection and in corpus luteum (CL) as compared to dominant follicles (DF) at day 5 of the estrous cycle (Fig. 1a; *P* < 0.0001). Additional experiments showed a time-dependent reduction in *JAK3* mRNA expression following hCG injection with the weakest expression observed after 24 h as compared to 0 h (Fig. 1b; *P* < 0.001). The endogenous luteinizing hormone (LH) surge model confirmed the downregulation of *JAK3* mRNA in the 24-h post-LH sample as compared to 0 h (Fig. 1c). Western blot analysis using anti-JAK3 antibodies confirmed a downregulation of JAK3 by hCG as the protein expression was significantly stronger in DF as compared to OF (Fig. 1d).

### Yeast two-hybrid screening revealed potential JAK3 partners in granulosa cells

The construct pGBKT7-JAK3 was not toxic to the Y2HGold yeast strain and JAK3 did not, by itself, activate the transcription of the reporter genes (*AUR-C*, *ADE2*, *HIS3*, and *MEL1*), since no colonies grew when Y2HGold[pGBKT7-JAK3] was plated in the presence of aureobasidin A antibiotic (data not shown). To verify that JAK3 protein was expressed in yeast cells, total proteins from Y2HGold strain transformed with pGBKT7-JAK3 was used to perform western blot analysis using anti-c-Myc antibodies. The western blot analysis confirmed JAK3 expression in the Y2HGold yeast strain (data not shown).

The mating of the Y2HGold[pGBKT7-JAK3] strain with the Y187[pGADT7-cDNA] strain resulted in the presence of zygotes that indicated a potential interaction between the bait (JAK3) and prey proteins contained in the granulosa cell library. Plasmids were purified from positive yeast colonies, amplified by PCR and sequenced. Sequence analyses resulted in different genes whose protein products have potential interactions with JAK3, including LEPROTL1, IHNBA and CDKN1B (Table 1).

**Table 1:** List of confirmed JAK3 partners in granulosa cells

Genes	Accession #^a^	Freq.	Ident. (%)	E-value	Description
INHBA	NM_174363.2	4	100	0.0	B.T. Inhibin, beta A
LEPROTL1	BT025351.1	2	100	1e-101	B.T. Leptin receptor overlapping transcript-like 1
CDKN1B	NM_001100346.1	3	99	3e-146	B.T. Cyclin-dependent kinase inhibitor 1B (p27, Kip1)

^a^Accession number of the best match found following nucleotide sequence comparison via BLAST search in GenBank.Freq., Frequency of cDNA clone identification from yeast two-hybrid prey library; Ident. (%), Identity: represents homology estimates ofbovine prey cDNA fragments with nucleotide sequences in GenBank database; B.T., *Bos taurus*

Sequences were analyzed for identity and resulted in candidate proteins potentially interacting with JAK3

Plasmids were purified from true positive yeast colonies, amplified by PCR and sequenced

### JAK3 physically interacts with LEPROTL1, INHBA, and CDKN1B

**Fig. 2:**
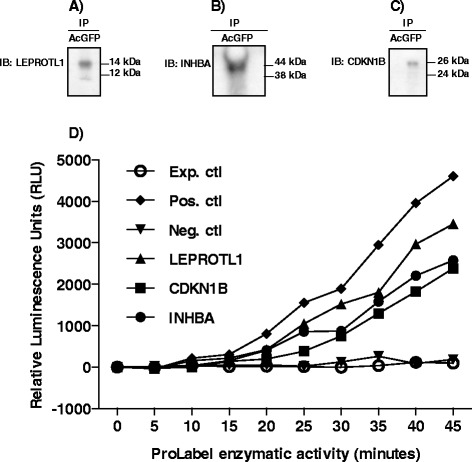
Confirmation of JAK3 interactions by co-immunoprecipitation, western blot analyses and luminescence measurement. LEPROTL1 (**a**), INHBA (**b**) and CDKN1B (**c**) were confirmed as physically interacting with JAK3. D) Relative luminescence was recorded as a result of ProLabel activity following interactions between JAK3 and LEPROTL1, INHBA and CDKN1B. There was a 42-fold, 34-fold and 15-fold induction in ProLabel enzymatic activity in HEK cells co-transfected, respectively, with JAK3 and LEPROTL1, JAK3 and INHBA, and JAK3 and CDKN1B as compared to the negative control. Exp. Ctl, experimental control; Pos. ctl, positive control; Neg. ctl, negative control

Physical interactions between JAK3 and candidate partners were confirmed by in vitro co-immunoprecipitation followed by western blot analyses and luminescence measurements. *JAK3* was cloned into pAcGFP1 vector while *LEPROTL1*, *INHBA* and *CDKN1B* were cloned, separately, into pProLabel vector. HEK 293 cells were then co-transfected with pAcGFP1-JAK3 and pProLabel-LEPROTL1, pProLabel-INHBA or pProLabel-CDKN1B. Following each co-transfection, cells were collected, proteins extracted and precipitated with anti-AcGFP antibodies. The protein complexes were pulled down using Protein G Plus/A Agarose Beads, subjected to SDS-PAGE and western blotting using anti-LEPROTL1, anti-INHBA or anti-CDKN1B. The results confirmed the presence of LEPROTL1 migrating at 14 kDa (Fig. 2a), INHBA at 44 kDa (Fig. 2b), and CDKN1B at 26 kDa (Fig. 2c) as interacting partners of JAK3. Additionally, relative luminescence was recorded as a result of ProLabel activity following interactions between JAK3 and LEPROTL1, INHBA or CDKN1B (Fig. 2d). Indeed, after 25 min of addition of the substrate, there was a 42-fold, 34-fold and 15-fold induction in ProLabel enzymatic activity in HEK cells co-transfected, respectively, with JAK3 and LEPROTL1, JAK3 and INHBA or JAK3 and CDKN1B as compared to the negative control (Fig. 2d).

### JAK3 partners are differently regulated during follicular development

**Fig. 3:**
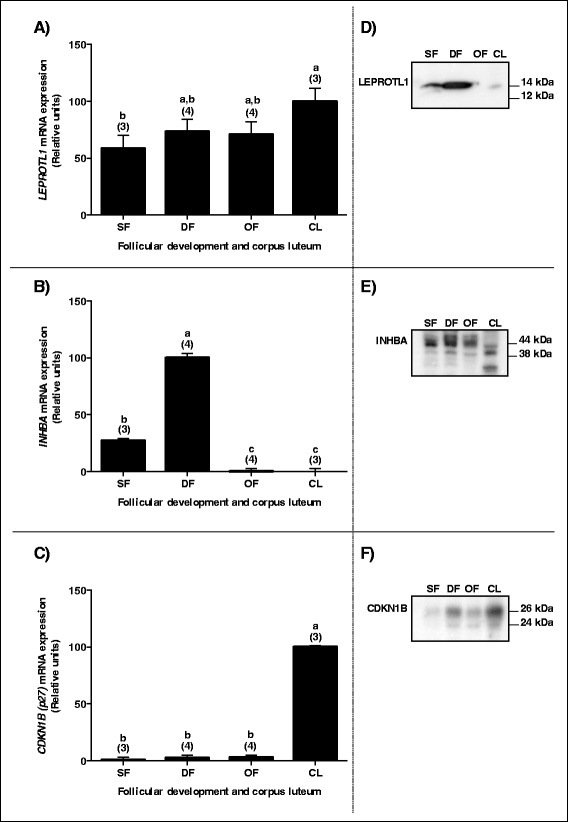
Regulation of JAK3 mRNA and protein partners during follicular development. Panels **a**), **b**), and **c**) Total RNA extracts of GC from SF, DF, OF, and CL were used to analyze *LEPROTL1*, *INHBA* and *CDKN1B* expression by real time RT-qPCR with *GAPDH* as reference gene. *LEPROTL1* mRNA expression was significantly increased in the CL as compared to SF (**a**) while there were no differences among the SF, DF and OF groups. Expression of *INHBA* was significantly greater in DF as compared to SF, OF and CL (**b**); expression of *INHBA* was also greater in SF than in OF and CL (**b**). For *CDKN1B*, mRNA expression was significantly greater in the CL as compared to SF, DF and OF (**c**)

Total RNA extracts of GC from small follicles (SF), dominant follicles (DF), ovulatory follicles (OF), and corpus luteum at day 5 of the estrous cycle (CL) were analyzed by RT-qPCR for *LEPROTL1*, *INHBA* and *CDKN1B* expression using specific primers. There was no significant difference in *LEPROTL1* mRNA expression throughout follicular development from small to ovulatory follicles (Fig. 3a), although *LEPROTL1* expression significantly increased in the CL as compared to SF. Expression of *INHBA* was significantly greater in DF as compared to SF, OF and CL (Fig. 3b). For *CDKN1B*, the steady-state mRNA level was greatest in the CL as compared to granulosa cells of SF, DF and OF, while there was no difference among the different groups of follicles (Fig. 3c).

Protein expression was analyzed for these partners and the results showed that LEPROTL1 protein expression was strongest in DF, nearly absent in OF and weak in the CL (Fig. 3d), despite the fact that there was no significant variation in *LEPROTL1* mRNA expression among the different groups of follicles. This result suggests a potential post-transcriptional regulation of LEPROTL1 activity in granulosa cells. Similar to its mRNA expression, INHBA protein was stronger in SF and DF as compared to OF and CL (Fig. 3e), while CDKN1B protein expression appeared strongest in the CL, moderate in DF and weak in SF and OF (Fig. 3f).

### STAT proteins in granulosa cells

**Fig. 4:**
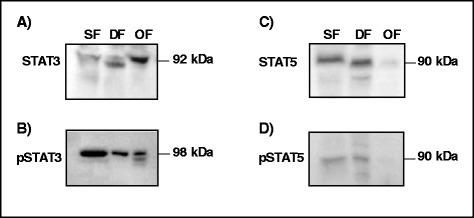
Expression of STAT3 and STAT5 proteins in granulosa cells during follicular development using samples from small (SF), dominant (DF), and ovulatory (OF) follicles. pSTAT, phospho STAT. STAT3 was expressed throughout follicular development (panel **a**) while pSTAT3 expression was stronger in SF and DF as compared to OF (panel **b**). The amounts of STAT5 (panel **c**) and pSTAT5 (panel **d**) were weaker in OF than in SF and DF

Western blot analyses using SF, DF, and OF samples showed expression of STAT3 in GC throughout follicular development (Fig. 4a). Although these analyses were not quantitative, expression of STAT3 appeared stronger in OF as compared to SF and DF. In contrast, expression of phospho-STAT3 (pSTAT3) was stronger in SF and DF as compared to OF (Fig. 4b). For STAT5, both the non-phosphorylated (Fig. 4c) and the phosphorylated (Fig. 4d) forms are present in SF and DF with no apparent differences between these two groups of follicles. However, expression of STAT5 and pSTAT5 was weaker in OF as compared to SF and DF (Fig. 4c and d).

### JAK3 participates in the phosphorylation of STAT3 in endometrial cells

**Fig. 5:**
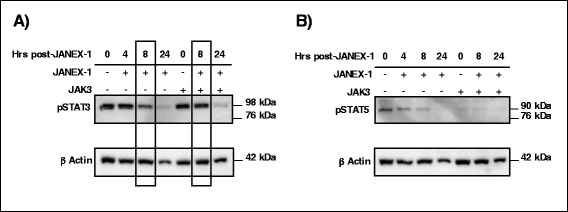
Phosphorylation assays of STAT3 and STAT5 by JAK3 in endometrial cells. Endometrial cells were put into culture and transfected or not with JAK3. Cells were then treated with 100 μM of JANEX-1 for 4, 8 and 24 h while control cells did not receive JANEX-1 treatment. Cells were collected using the M-PER buffer and protein extracts were subjected to western blot analyses. A) JANEX-1 reduced the phosphorylation of STAT3 after 8 h of treatment and more so after 24 h, while transfection with JAK3 prior to JANEX-1 treatment increased the amount of pSTAT3. B) JANEX-1 reduced the amount of pSTAT5 in endometrial cells not transfected with JAK3 while transfection with JAK3 considerably reduced the amount of pSTAT5 as compared to non-transfected cells

Bovine endometrial cells were cultured and treated with or without JANEX-1 and in the presence or absence of JAK3. The addition of JANEX-1 reduced phosphorylation of STAT3 after 8 h of treatment and more so after 24 h (Fig. 5a). When cells were transfected with JAK3 prior to JANEX-1 treatment, the amount of pSTAT3 was stronger as compared to non-transfected cells after 8 h post-JANEX-1 treatment (Fig. 5a, boxed lanes), meaning that JAK3 increased STAT3 phosphorylation, which was then reduced by JANEX-1 after 24 h (Fig. 5a). Similarly, the addition of JANEX-1 reduced the amount of phosphorylated STAT5 in endometrial cells not transfected with JAK3 (Fig. 5b). The reduction of pSTAT5 amount was observed at 4 h post-JANEX-1 treatment, and after 24 h, pSTAT5 was nearly extinct in the cells (Fig. 5b). Surprisingly, overexpression of JAK3 by transfection in endometrial cells considerably reduced the amount of phosphorylated STAT5 as compared to non-transfected cells (Fig. 5b). The addition of JANEX-1 did not have any effects on pSTAT5 amounts in JAK3-transfected cells.

### JAK3 expression increased endometrial cell viability

**Fig. 6:**
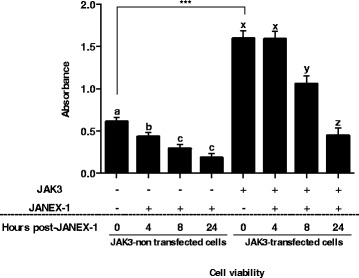
Comparison of cell viability on cultured endometrial cells treated with JANEX-1. In JAK3-non transfected cells, treatment with JANEX-1 (100 μM) significantly reduced the number of viable cells from 4 h through 24 h as compared to the control (*P* < 0.0002). In JAK3-transfected cells, significant reduction of cell viability by JANEX-1 was observed from 8 h after treatment (*P* < 0.0001). JAK3 overexpression in endometrial cells significantly increased cell viability as compared to non-transfected cells (*P* < 0.0004). Means and standard error of means (SEM) are shown

To assess the role of JAK3 in cell viability, endometrial cells were cultured and treated with or without JANEX-1, and in the presence or absence of JAK3 overexpression. Cell viability measurements showed a significantly higher proportion of viable cells prior to treatments with JANEX-1 (Fig. 6). In JAK3-non transfected cells, cell viability was significantly reduced within 4 h of JANEX-1 treatment (*P* < 0.0002), while in JAK3-transfected cells, a significant reduction in cell viability was observed from 8 h post JANEX-1 treatment (*P* < 0.0001). In the absence of JANEX-1, JAK3 significantly increased cell viability as compared to non-transfected cells (Fig. 6, *P* < 0.0004).

## Discussion

We report for the first time JAK3 regulation and protein interactions in the reproductive system using granulosa cells from ovarian follicles. We also demonstrated that a functional JAK3 affects phosphorylation of STAT proteins suggesting that JAK/STAT signaling is operating in these bovine reproductive cells. The major findings of this study are that: 1) the greatest expression of JAK3 is associated with the growing, estrogen-active follicle; 2) JAK3 expression is hormonally-regulated as it is significantly reduced by the endogenous LH surge and by hCG injection in a time-dependent manner, and in the corpus luteum after granulosa cells differentiation into luteal cells; and 3) JAK3 phosphorylates STAT3 in endometrial cells and increases cell viability since inhibition of JAK3 with JANEX-1 significantly reduces both the phosphorylation of STAT3 and cell viability. These observations indicate that JAK3 is associated with increased cell proliferation. It is known that JAK activation and the JAK/STAT pathway regulate cell proliferation, differentiation, migration and apoptosis depending on the signal, the tissue, and the cellular context [10, 34, 35]. Because JAK3 expression in the OF and CL is considerably reduced compared to the growing follicle, it is conceivable that JAK3 might be involved in granulosa cell proliferation rather than in their differentiation.

Members of the JAK and STAT families constitute a crucial signaling system that has been the focus of extensive studies, notably in the function of immune cells. JAK3 is primarly expressed in leukocytes and is required for their development and function [36]. JAK3 typically associates with the common γc subunit of cytokine receptors, becomes activated following cytokine binding to the receptor [6], and phosphorylates other proteins, including STAT proteins, that mediate gene transcription [37]. The range of proteins activated specifically by JAK3 as well as the protein-protein interactions in which JAK3 is involved could mediate different mechanisms in various systems. JAK3 has been shown to phosphorylate insulin receptor substrate-1 (IRS-1), IRS-2, PI3K/Akt and focal adhesion kinase (FAK) [38], which contain SH2 or other phospho-tyrosine-binding domains. Other studies have used Drosophila to investigate the JAK/STAT pathway in ovarian cell migration, sex determination and stem-cell maintenance [39, 40], and in macrophages infected with *F. tularensis,* JAK3 is involved in the phosphorylation of p38MAPK [41]. Herein, we observed that JAK3 strongest expression was found in the dominant follicle, which is active and growing while in the ovulatory follicle, following the endogenous LH surge or hCG injection, JAK3 expression was dramatically reduced. These results suggest a specific role for JAK3 in granulosa cells during follicular growth between the stages of small antral follicles to preovulatory follicles, prior to the LH surge, such as in granulosa cells proliferation through the activation or inhibition of target proteins.

Using a yeast two-hybrid screening, we identified and confirmed novel JAK3-interacting proteins in granulosa cells that can act as downstream target proteins for JAK3 signaling within the follicle. Some of these partners are known to be expressed in dominant follicles and participate actively in follicular development. Of interest, we showed that inhibin beta A (INHBA) interacts with JAK3 in granulosa cells of dominant follicles. Previous spatiotemporal expression studies showed greatest mRNA expression for *INHBA* in estrogen active follicles [42–44] and an increase in activin-A protein secreted in follicular fluid [45]. Activin promotes granulosa cells proliferation and steroidogenesis and potentiates FSH actions on granulosa cells by increasing *FSH receptor* expression [46, 47], which underscores a key role for activin-A in the dominant follicle’s development. Our data confirmed expression of *INHBA* mRNA and protein in dominant follicles in agreement with already published data. The interaction between JAK3 and INHBA could increase granulosa cells proliferation and participate in the growth of the dominant follicle into the ovulatory stage, prior to the LH surge.

In addition to INHBA, physical interactions were confirmed by co-IP between JAK3 and potential partners including LEPROTL1 and CDKN1B. Leptin receptor overlapping transcript-like 1 (LEPROTL1), also referred to as endospanin 2 [48], is a transmembrane protein involved in the regulation of intracellular protein trafficking [49]. It belongs to the OB-RGRP/VPS55 family and is widely expressed [50]. LEPROTL1 was isolated from a human fetal brain cDNA library and was shown to possess a JAK binding site (Pro(46)-Ile-Pro(48)) [50]. LEPROTL1 negatively regulates growth hormone (GH) receptor cell surface expression in liver and may play a role in liver resistance to GH during periods of reduced nutrient availability [51]. Functionally, it has been shown that transgenic mice overexpressing LEPROTL1 displayed growth retardation and an impairment of GH-induced STAT5 phosphorylation in the liver [51]. These studies indicate that LEPROTL1 expression decreased GH signaling while LEPROTL1 silencing increased GH signaling. Furthermore, recent studies demonstrated that fibroblast growth factor 21 (FGF21) inhibition of GH stimulatory actions on IGF-1 expression in chondrocytes depends on the intracellular activity of LEPROTL1 and LEPROT, another member of the family [49]. Based on the available data and our findings, one could assume that JAK3 binding to LEPROTL1 may participate in the follicular growth process by reducing the potential negative feedback of LEPROTL1 on the GH signaling and increasing IGF-1 availability in the growing follicle. In this regard, it has been shown that GH increased IGF-1 secretion by ovine granulosa cells *in vitro* [52] supporting the idea that an increased GH signaling may regulate the ovarian function through IGF-1 production by granulosa cells.

The third JAK3 partner that was confirmed by co-IP was the cyclin-dependent kinase inhibitor 1B (CDKN1B or p27(KIP1)). CDKN1B is a cyclin-dependent kinase inhibitor that binds to and prevents the activation of cyclin E-CDK2 or cyclin D-CDK4 complexes, and thus controls the cell cycle progression at G1 [53, 54]. The degradation of CDKN1B, which is triggered by its CDK-dependent phosphorylation and subsequent ubiquitination, is required for the cellular transition from quiescence to the proliferative state [55]. CDKN1B acts either as an inhibitor or an activator of cyclin type D-CDK4 complexes depending on its phosphorylation state and/or stoichiometry. The phosphorylation of CDKN1B occurs on serine, threonine and tyrosine residues. Phosphorylation on Ser-10 is the major site of phosphorylation in resting cells and takes place at the G(0)-G(1) phase leading to protein stability [56]. Phosphorylation on other sites is greatly enhanced by mitogens, growth factors, cMYC and in certain cancer cell lines [57, 58]. As a result, the phosphorylated CDKN1B form found in the cytoplasm is inactive. Based on these reports, and in light of our current findings, it may be possible that CDKN1B binding to JAK3 leads to its phosphorylation and inactivation in granulosa cells of dominant follicle, thus allowing the progression of granulosa cell division and proliferation. Indeed, the greatest expression of CDKN1B in the corpus luteum suggests a role for CDKN1B in establishing the nonproliferative state, which is required for differentiation or for proper functioning of the differentiated luteal cells. Our findings are consistent with previous results showing that the luteinization process is associated with up-regulation of *CDKN1B (p27)* that accumulated during initial phases of luteinization and remained elevated until termination of the luteal function [59]. Our data are also consistent with the report that, in the bovine ovary, the expression of *CDKN1B (p27)* mRNA was significantly reduced in granulosa cells from oestrogen-active dominant follicles as compared to oestrogen-inactive follicles [60], meaning that cyclins and their inhibitors are associated with granulosa cell proliferation at specific follicular developmental stages.

Because of the difficulty of obtaining and culturing bovine primary granulosa cells from dominant follicles, we used a bovine endometrial cell line to demonstrate that JAK3 increased phosphorylation of STAT3 and that addition of JANEX-1, a specific JAK3 inhibitor, reduced the amount of JAK3-phosphorylated STAT3. These results suggest that the phosphorylation of STAT3 is directly linked to the presence of JAK3 in these cells. Moreover, the presence of JAK3 was also associated with increased cell viability since JANEX-1 significantly reduced the proportion of viable cells while overexpression of JAK3 delayed the decrease in cell viability induced by JANEX-1. Together, these data suggest the presence of a functional JAK3 and possibly JAK/STAT pathway in endometrial cells that may be involved in cell proliferation. During follicular development, JAK3 is predominantly expressed in the growing dominant follicle, suggesting that JAK3 may impact granulosa cell proliferation through the phosphorylation of target proteins including STAT transcription factors. We have shown that levels of phosphorylated STAT3 was stronger in small and dominant follicles as compared to ovulatory follicles and even stronger in small compared to dominant follicles. These observations could mean that STAT3 phosphorylation by JAK3 in granulosa cells may be associated with follicular growth. However, it was recently proposed that the stronger amount of pSTAT3 in small follicles was associated with granulosa cells death and follicular atresia [61]. It would be relevant to verify whether JAK3 increases STAT3 phosphorylation in bovine granulosa cells of atretic small follicles as compared to healthy growing follicles. Although we have demonstrated the presence of pSTAT3 in granulosa cells and an increased STAT3 phosphorylation by JAK3 in endometrial cells, further functional studies should clarify the roles of pSTAT3 in granulosa cell death in small follicles and in granulosa cell proliferation in the growing dominant follicles following a potential activation by JAK3. As for STAT5, surprisingly, JAK3 overexpression seemed to reduce the amount of pSTAT5 in endometrial cells transfected with JAK3 although pSTAT5 is detected in non-transfected cells and that JANEX-1 reduced pSTAT5 amounts. These findings could mean that basal expression of JAK3 is required for STAT5 phosphorylation while overexpression of JAK3 leads to the inhibition of STAT5 phosphorylation.

## Conclusions

This study demonstrated that JAK3 is hormonally regulated in bovine granulosa cells and identified partners to JAK3 including LEPROTL1, INHBA and CDKN1B whose binding to JAK3 may participate in follicular growth through their activation or inhibition by JAK3. Using a JAK3 inhibitor, we showed that JAK3 increased phosphorylation of STAT3 but decreased phosphorylation of STAT5 and was associated with increased cell viability. Together, these results support a role for JAK3 in follicular growth through increased cell viability and kinase activity and contribute to expand our understanding of the activity and control of the ovary.

## Methods

### Cloning and characterization of JAK3

*JAK3* was characterized from a differential hybridization screening of a subtracted cDNA library using the suppression subtractive hybridization approach [30]. We established and analyzed a granulosa cell-cDNA library from dominant preovulatory follicles and the cDNA clone corresponding to JAK3 was identified as differentially expressed in these follicles. JAK3 cDNA was amplified by PCR and entirely characterized by sequencing before proceeding to subsequent experiments.

### Experimental animal model and sample preparations

The regulation of JAK3 expression during follicular development was studied using *in vivo* models as previously characterized [30]. Following estrous synchronization with PGF2α, normal cycling crossbred heifers were randomly assigned to a dominant follicle group (DF; *n* = 4), or an ovulatory hCG-induced follicle group (OF; *n* = 4). In the DF group, the ovary bearing the DF on the morning of day 5 of the estrous cycle (day 0 = day of estrus) was obtained by ovariectomy (via colpotomy). The DF was defined as being > 8 mm in diameter and growing while subordinate follicles were either static or regressing. The OF were obtained following an injection of 25 mg of PGF2α on day 7 to induce luteolysis, thereby promoting the development of the DF of the first follicular wave into a preovulatory follicle. An ovulatory dose of hCG (3000 IU, iv; APL, Ayerst Lab, Montréal, QC) was injected 36 h after the induction of luteolysis, and ovaries bearing the hCG-induced OF were collected by ovariectomy 24 h post-hCG. Additional OF were collected at 0, 6, 12, 18, and 24 h after hCG injection for follicular wall preparation (*n* = 2 cows/time point). The sample at 0 h was represented by day 7 dominant follicle. Immediately following ovariectomy, follicles were dissected into preparations of follicular wall (theca interna with the attached granulosa cells) [62] or further dissected into separate isolates of granulosa cells [30], and stored at –70 °C. Additionally, granulosa cells (GC) were collected from 2 to 4 mm small follicles (SF) obtained from slaughterhouse ovaries, and a total of three pools of twenty SF were prepared. Concentrations of progesterone (P_4_) and estradiol-17β (E_2_) as well as their ratio (P_4_/E_2_) were validated by radioimmunoassay of follicular fluid. Corpora lutea (CL) at day 5 of the estrous cycle were obtained by ovariectomy and were dissected from the ovarian stroma, frozen in liquid nitrogen, and stored at –70 °C. All animal procedures were approved by the Animal Ethics Committee of the Faculty of Veterinary Medicine of the University of Montreal.

### mRNA expression analysis

Expression and regulation of *JAK3* mRNA during follicular development and following hCG injection was analyzed by semi-quantitative RT-PCR. Total RNA was extracted from bovine GC collected from follicles at different developmental stages (SF, DF, OF) and CL, and from the follicular walls (granulosa and theca cells) collected at 0, 6, 12, 18, and 24 h post-hCG injection. Specific *JAK3* PCR primers (Table 2) were used and the number of cycles was limited and optimized for analysis of *JAK3* mRNA expression. PCR reaction products were separated on a 1.2 % TAE-agarose gel with ethidium bromide, visualized by UV light, digitized and analyzed by densitometry using ImageQuant software (GE Healthcare Life Sciences). *GAPDH* was used as a reference gene, and specific signals of *JAK3* were normalized with corresponding *GAPDH* signals.

**Table 2:** Primers used in the expression analyses of *Bos taurus* genes by PCR and RT-qPCR

Gene		Primer sequence (5′–3′)^a^	Accesion no.	AS (bp)
GAPDH	Fwd	tgttccagtatgattccacccacg	NM_001034034	703
	Rv	ggttgtctcctgcgacttcaacag		
INHBA	Fwd	gctcatccctctcctttcact	NM_174363	171
	Rv	cgatcatgttctggatgtcct		
LEPROTL1	Fwd	tactggcccctctttgttctg	NM_001037462	207
	Rv	caagctccccactcgatcaaa		
CDKN1B	Fwd	tgtcaaacgtgcgagtgtcta	NM_001100346	150
	Rv	ctctgcagtgcttctccaagt		
JAK3	Fwd	gcacctccaagtgaggagac	XM_010806603	729
	Rv	cctctcgaggtccatgatgt		

*Abbreviations*: *AS* amplicon size (base pairs), *Fwd* forward primer, *Rv*, reverse primer

^a^All primers were designed and validated by the authors. Each primer was used at a final concentration of 600 nM

### Cell extracts and immunoblotting analysis

Granulosa cells and CL were obtained as described above. Tissue samples were homogenized in T-PER buffer (Pierce, Rockford, IL, USA) supplemented with complete protease inhibitors (Roche Diagnostics, Laval, QC, Canada), and centrifuged at 16,000 × *g* for 10 min at 4 °C. The recovered supernatant was stored at −70 °C until electrophoretic analyses were performed. For cell cultures, the M-PER mammalian protein extraction reagent (Pierce) was used. M-PER reagent was added to the plates, cell debris was removed by centrifugation (14,000 × g for 5 min at 4 °C) and the supernatants were collected and stored at −70 °C until further analyses. Total protein concentrations were determined using the Bradford method [63] (Bio-Rad Protein Assay, Bio-Rad Lab, Mississauga, ON, Canada) and immunoblotting was performed as described previously [64]. Samples (50 μg of proteins) were resolved by one-dimensional denaturing 12 % Novex Tris-glycine gels (Invitrogen, Burlington, ON, Canada) and transferred onto polyvinylidene difluoride membranes (PVDF; GE Healthcare Life Sciences). Membranes were incubated with specific first antibodies against JAK3, STAT3, phospho-STAT3 (pSTAT3), STAT5, and phospho-STAT5 (pSTAT5). All antibodies and corresponding concentrations are listed in Table 3. Immunoreactive proteins were visualized by incubation with appropriate horseradish peroxidase-linked secondary antibodies (1:10 000 dilution) followed by incubation with the enhanced chemiluminescence system, ECL plus (GE Healthcare Life Sciences) according to the manufacturer’s protocol and revelation using the ChemiDoc XRS+ system (Bio-Rad).

**Table 3:** Antibodies used in the expression analyses of *Bos taurus* proteins by Western blot

Target proteins	Antibodies	Sources	MW (kDa) Conc.	
Beta Actin	Chicken IgY anti-Beta actin antibody	Immune Biosolutions (Cat. # Y00005-002)	42	---
JAK3	Goat polyclonal anti-JAK3	Santa Cruz Biotechnology (Cat. # sc-1080)	125	0.4 μg/ml
STAT3	Rabbit monoclonal anti-STAT3	Abcam (Cat. # ab32500)	92	0.069 μg/ml
pSTAT3	Rabbit monoclonal anti-pSTAT3	Abcam (Cat. # ab32143)	98	1 μg/ml
STAT5	Rabbit polyclonal anti-STAT5	Abcam (Cat. # ab68465)	90	0.5 μg/ml
pSTAT5	Rabbit polyclonal anti-pSTAT5	Abcam (Cat. # ab97734)	90	1 μg/ml
LEPROTL1	Rabbit polyclonal anti-LEPROTL1	Abcam (Cat. # ab139564)	14	1 μg/ml
INHBA	Rabbit polyclonal anti-INHBA	Aviva Systems Biology (# ARP54663_P050)	44	0.5 μg/ml
CDKN1B	Goat polyclonal anti-CDKN1B/KIP1	Aviva Systems Biology (# OAEB01630)	26	0.1 μg/ml

*Abbreviations*: *pSTAT*, phospho STAT, *MW* molecular weight of target proteins analyzed, *Conc*., antibody concentrations used for immunoblotting

### Yeast two-hybrid procedure

#### JAK3 constructs for Y2H bait preparation

A detailed description of the yeast two-hybrid procedure used in this study is provided in the Additional file 1. Briefly, a JAK3 construct was generated by PCR amplification of a 729 bp-fragment corresponding to the FERM domain of JAK3 localized in the amino terminus region. The PCR product was purified and cloned in frame with the GAL4-DNA binding domain into the pGBKT7 vector to produce a bait plasmid using the Matchmaker Gold Yeast Two-Hybrid System (Clontech) exactly as proposed by the manufacturer. The bait plasmid (pGBKT7-JAK3) was used to transform Y2HGold yeast strains using the Yeast Transformation system 2 kit (Clontech) and this bait was referred to as Y2HGold[pGBKT7-JAK3]. Y2HGold yeast cells harbor four reporter genes (*HIS3*, *ADE2*, *MEL1* and *AUR1*) under the control of *GAL4* upstream activating sequences, which are used to detect two-hybrid interactions. To determine whether JAK3 protein was expressed, c-Myc monoclonal antibody was used in western blot analysis to detect JAK3 protein in yeast cells containing pGBKT7-JAK3 constructs. To confirm that the pGBKT7-JAK3 bait did not autonomously activate the reporter genes in Y2HGold in the absence of a prey protein, competent Y2HGold cells were transformed with pGBKT7-JAK3 and the transformant were plated on appropriate selective agar plates (See Additional file 1). In parallel, competent Y2HGold cells were transformed with pGBKT7 empty vector (pGBKT7) along with pGBKT7-JAK3 to verify toxicity of the JAK3 bait protein when expressed in yeast cells. All plated yeast cells were incubated at 30 °C for 5 days.

#### Generation of granulosa cells (GC)-cDNA library and construction of the two-hybrid prey library

A bovine GC-cDNA prey library from dominant follicles was prepared in the Y187 yeast strain using the pGADT7-Rec vector. cDNAs were expressed and fused to the GAL4 activating domain using the Matchmaker library construction & screening kit (Clontech) as detailed by the manufacturer. Total RNA was isolated from GC of dominant follicles and used to generate cDNAs. Oligo dT (CDSIII)-primed cDNAs were generated using the Make Your Own “Mate & Plate” Library System (Clontech) with 1 μg of bovine GC RNA. Long distance PCR (LD-PCR) was performed using the Advantage 2 Polymerase Mix, PCR products were purified (size-selected) using CHROMA SPIN + TE-400 columns (Clontech), and the resulting cDNAs were analyzed on a 1 % agarose gel. To create the prey library, competent Y187 yeast cells were prepared and co-transformed with 3 μg of dominant follicle GC-cDNAs and the pGADT7-Rec plasmid (0.5 μg/μl). The co-transformed yeast cells were spread on 150 mm selective agar plates. After 5 days of incubation at 30 °C, the 150 mm plates were chilled at 4 °C for 3 h and 5 ml of freezing medium was added to each plate to harvest and pool all transformants. Cell density was estimated using a hemocytometer and the library was frozen at −70 °C. This library was referred to as Y187[pGADT7-cDNA].

#### Screening of the two-hybrid library using yeast mating

A detailed description of the screening procedure is provided in the Additional file 1. Briefly, Y2HGold yeast cells carrying the bait plasmids (Y2HGold[pGBKT7-JAK3]) were mated with Y187 yeast cells harboring the bovine GC-cDNA library (Y187[pGADT7-GC]). Only library plasmids responsible for the activation of all four reporter genes were rescued and isolated using the Easy yeast plasmid isolation kit (Clontech). To generate sufficient plasmid to allow their characterization by sequencing, prey plasmids were used to transform Stellar competent bacteria that were plated on LB agar plates with 100 μg/ml of ampicillin. The prey inserts were identified by sequencing and nucleic acid sequences were verified for the presence of an open reading frame fused in frame to the *GAL4* AD sequence. Prey insert sequences were compared to GenBank sequences via BLAST analysis to determine their identities.

### Co-IP confirmation of protein interactions

Physical interaction between JAK3 and candidate partners was confirmed by *in vitro* co-immunoprecipitation assay using the Matchmaker Co-IP system (Clontech). The JAK3 bait was cloned into the linearized pAcGFP1-C vector and its potential prey partners from the GC-cDNA library were cloned into the linearized pProLabel-C vector using the In-fusion HD EcoDry cloning kit (Clontech). The two plasmid constructs were used to co-transfect HEK 293 cells using the CalPhos Mammalian transfection kit (Clontech) as recommended by the manufacturer. Fourthy-eight hours post-transfection, cells were collected and cell lysates were prepared for the co-IP assay. Each lysate sample was incubated with 1 μl of the anti-AcGFP polyclonal antibody on a rotator at 4 °C for 2 h. After the antibody incubation, the entire volume of each sample was transferred to a tube containing washed Protein G Plus/Protein A Agarose Beads and incubated overnight at 4 °C with gentle rotation. Beads were thereafter gently pelleted by centrifugation at 4 °C, 5000 × g for 10 s followed by washing. The samples were subjected to SDS-PAGE analysis and blotted with specific prey antibodies to confirm the presence of prey proteins physically interacting with JAK3 using specific antibodies (Table 3). The selected preys were leptin receptor overlapping transcript-like 1 (LEPROTL1), inhibin beta A (INHBA), and cyclin-dependent kinase inhibitor 1B (CDKN1B) based on the number of times they were identified and their potential (for LEPROTL1 and CDKN1B) or known (INHBA) roles in the ovary. In addition, the luminescence generated as ProLabel enzymatic activity was measured in protein samples from cells co-transfected with pAcGFP1-JAK3 and separate prey partners along with appropriate controls using the ProLabel detection kit (Clontech) following the manufacturer’s protocol.

### Regulation of JAK3 partners during follicular development

The regulation of JAK3 partners expression were analyzed during follicular development at the mRNA and protein levels using, respectively, RNA samples from SF, DF, OF and CL, and protein samples from granulosa cells of SF, DF and OF as described above. Expression of mRNA for *INHBA*, *LEPROTL1*, and *CDKN1B* was quantified by real time RT-qPCR using specific primers (Table 2) and the results were analyzed using the Livak method (2^-ΔΔCq^) [65]. Protein expression for INHBA, LEPROTL1 and CDKN1B was analyzed using corresponding antibodies (Table 3).

### JAK3 functional studies

To further study JAK3 function and determine whether its activity is necessary for the phosphorylation of target proteins, a bovine endometrial cell line (Endo 8.3) [66] were cultured and treated with or without JANEX-1, a JAK3 inhibitor [67], to verify for JAK3-phosphorylated STAT proteins. Endometrial cells were first transfected, or not, with the entire JAK3 open reading frame cloned into the pAcGFP1 vector using the CalPhos transfection kit (Clontech). JANEX-1 treatment (100 μM) was then added 16 h after JAK3 transfection. Whole cell lysates were collected at different time points after JANEX-1 treatment and subjected to western blot analysis to verify phosphorylation of STAT3 and STAT5 using, respectively, anti-phospho STAT3 and anti-phospho STAT5 antibodies. In addition, to determine cell viability using the CellTiter assay kit (Promega), endometrial cells were seeded on 96-well plates and incubated at 37 °C/5 % CO_2_ in DMEM supplemented with 10 % fetal bovine serum. After incubation with or without JANEX-1, the CellTiter substrate was added for 3 h before measuring absorbance at 490 nm with the SpectraMax i3 (Molecular Devices).

### Statistical analyses

Values for *JAK3* and other target gene mRNAs were normalized with those of the control gene *GAPDH*. Homogeneity of variance between groups was verified by O’Brien and Brown-Forsythe tests. Corrected values of gene specific mRNA levels were compared between follicular or CL groups by one-way ANOVA. When ANOVA indicated a significant difference (*P* < 0.05), the Tukey-Kramer test was used for multiple comparison of individual means among SF, DF, OF and CL, whereas the Dunnett test (*P* < 0.05) was used to compare different time points after hCG with 0 h as control. These statistical analyses were performed using JMP software (SAS Institute, Inc.). Statistical analyses for proliferation assays were performed using Prism software 6.0 for Macintosh (GraphPad). All data were presented as least-square means ± SEM.

## Additional file

Additional file 1: Detailed description of the Yeast two-hybrid procedure. (DOCX 114 kb)

## Abbreviations

CDKN1B: Cyclin-dependent kinase inhibitor 1B
DF: Dominant follicle
FAK: Focal adhesion kinase
FERM: 4.1, ezrin, radixin, moesin
GC: Granulosa cells
hCG: Human chorionic gonadotropin
INHBA: Inhibin beta A
IRS-1: Insulin receptor substrate-1
JAK3: Janus kinase 3
JH: JAK homology
LEPROTL1: Leptin receptor overlapping transcript-like 1
OF: Ovulatory follicle
P38MAPK: p38 mitogen-activated protein kinase
SF: Small follicle
STAT: Signal transducers and activators of transcription
