# Supplementary material for: Differential regulation of Janus kinase 3 (JAK3) in bovine preovulatory follicles and identification of JAK3 interacting proteins in granulosa cells
Source: J Ovarian Res. 2016 Oct 28;9:71. doi: 10.1186/s13048-016-0280-5 (PMC5086056; doi:10.1186/s13048-016-0280-5)
Supplement: Additional file 1: — Detailed description of the Yeast two-hybrid procedure. (DOCX 114 kb) [file 13048_2016_280_MOESM1_ESM.docx]

***Yeast two-hybrid (Y2H) procedure***

***Material and Media legend***

The following media and material were used for the yeast two-hybrid experiments, all of which were purchased from Clontech: YPD = Yeast extract, peptone, and dextrose; YPDA = YPD medium supplemented with adenine hemisulfate. SD = Minimal, synthetically defined medium for yeasts, which includes a nitrogen base, a carbon source (glucose), and a dropout (DO) supplement; DO = dropout solution: a mixture of specific amino acids and nucleosides used to supplement SD base. DO solutions are missing one or more of the nutrients required by untransformed yeast to grow on SD medium; DDO or SD/-Leu/-Trp = double dropout medium that includes every essential amino acid except for leucine and tryptophan, which are omitted. SD/–Leu/–Trp dropout supplement is used to select for bait and prey plasmids. Cells harboring bait and prey plasmids are able to grow because the plasmids encode tryptophan and leucine biosynthesis genes, respectively, that are otherwise absent from the cell; X = X-α-Gal; A or AbA = Aureobasidin A antibiotic; DDO/X/A = Double dropout medium (SD/-Leu/-Trp) supplemented with X-α-Gal and Aureobasidin A; QDO/X/A = Quadruple dropout medium (SD/-Leu/-Trp/-Ade/-His) lacking the four amino acids leucine, tryptophan, adenine, and histidine and supplemented with X-α-Gal and Aureobasidin A. QDO/X/A medium is used to select for the bait and prey plasmids, and to verify for the activation of the Gal-responsive *HIS3* and *ADE2* reporter genes as part of the confirmation step of the two-hybrid assay. Yeast colonies that grow on this quadruple dropout medium contain both the bait and prey plasmids and also express proteins that interact with each other to activate reporter genes *HIS3* and *ADE2*. Kanamycin sulfate was added to all yeast media at a final concentration of 50 µg/ml to prevent bacterial contamination.

***JAK3 constructs for Y2H bait preparation***

A JAK3 construct was generated by PCR amplification of a 729 bp-fragment corresponding to the FERM domain of JAK3 localized in the amino terminus region. The PCR product was purified and cloned in frame with the GAL4-DNA binding domain into the pGBKT7 vector to produce a bait plasmid using the Matchmaker Gold Yeast Two-Hybrid System (Clontech) exactly as proposed by the manufacturer. The bait plasmid (pGBKT7-JAK3) was used to transform Y2HGold yeast strains following the Yeast Transformation system 2 kit (Clontech) and this bait was referred to as Y2HGold[pGBKT7-JAK3]. Y2HGold yeast cells harbor four reporter genes (*HIS3*, *ADE2*, *MEL1* and *AUR1*) under the control of *GAL4* upstream activating sequences, which are used to detect two-hybrid interactions. To determine whether JAK3 protein was well expressed in yeast cells, c-Myc monoclonal antibody was used in western blot analysis to detect JAK3 protein in yeast cells containing pGBKT7-JAK3 constructs. To confirm that the pGBKT7-JAK3 bait did not autonomously activate the reporter genes in Y2HGold in the absence of a prey protein, competent Y2HGold cells were transformed with pGBKT7-JAK3 and the transformant (1/10 and 1/100 dilutions) were plated on SD/-Trp, SD/-Trp/X-α-Gal and SD/-Trp/X-α-Gal/AbA agar plates. In parallel, competent Y2HGold cells were transformed with pGBKT7 empty vector (pGBKT7) to demonstrate that the JAK3 bait protein was not toxic when expressed in yeast cells. These transformants were plated on SD/-Trp agar plates using 1/10 and 1/100 dilutions. A transformation control using pGBT9 (Clontech) was performed and plated on SD/-Trp plates to be compared to pGBKT7 and pGBKT7-JAK3 plates. All plated yeast cells were incubated at 30°C for 5 days.

***Generation of granulosa cells (GC)-cDNA library and construction of the two-hybrid prey library***

A bovine GC-cDNA prey library from dominant follicles was prepared in the Y187 yeast strain using the pGADT7-Rec vector. cDNAs were expressed fused to the GAL4 activating domain using the Matchmaker library construction & screening kit (Clontech) as detailed by the manufacturer. Total RNA was isolated from GC of dominant follicles and used to generate cDNAs. Oligo dT (CDSIII)-primed cDNAs were generated using the Make Your Own “Mate & Plate” Library System (Clontech). Briefly, cDNA synthesis was carried out with 1 μg of bovine GC RNA. Long distance PCR (LD-PCR) was performed using the Advantage 2 Polymerase Mix and PCR products were purified (size-selected) using CHROMA SPIN+TE-400 columns (Clontech). The resulting cDNAs were analyzed on a 1% agarose gel. To create the prey library, competent Y187 yeast cells were prepared and co-transformed with 3 µg of dominant follicle GC-cDNAs and the pGADT7-Rec plasmid (0.5 µg/µl). The co-transformed yeast cells were spread on 100 mm SD/-Leu agar plates (100 µl of 1/10 and 1/100 dilutions) to determine the number of independent clones in the library. The remainder of the transformed yeast cells was spread on 150 mm SD/-Leu agar plates. After 5 days of incubation at 30°C, the 150 mm plates were chilled at 4°C for 3 hours and 5 ml of freezing medium (YPDA+25% glycerol) was added to each plate to harvest and pool all transformants. Cell density was estimated using a hemocytometer and the library was frozen at -70°C. This library was referred to as Y187[pGADT7-cDNA].

***Two-hybrid library screening using yeast mating***

Control experiments were conducted before screening of the two-hybrid Y187[pGADT7-cDNA] library. A set of positive and negative controls was performed using the constructs pGBKT7-53 with pGADT7-T as positive control and pGBKT7-Lam with pGADT7-T as negative control. pGBKT7-53 encodes the Gal4 DNA-BD fused with murine p53; pGADT7-T encodes the GAL4 AD fused with SV40 large T-antigen. Since p53 and large T-antigen are known to interact in a yeast two-hybrid assay [30, 31], mating Y2HGold[pGBKT7-53] with Y187[pGADT7-T] will result in diploid cells containing both plasmids that can activate all four reporters. Both positive and negative control matings were spread on SD/-Leu, SD/-Trp, DDO and DDO/X/A agar plates using 1/10 and 1/100 dilutions from the mated cultures. Diploid yeast cells containing pGBKT7-Lam and pGADT7-T grew on SD/–Leu, SD/–Trp and SD/–Leu/–Trp (DDO) minimal media, but no colonies grew on DDO/X/A.

To screen for protein partners, Y2HGold yeast cells carrying the bait plasmids (Y2HGold[pGBKT7-JAK3]) were mated to Y187 yeast harboring the bovine GC-cDNA library (Y187[pGADT7-GC]). A concentrated overnight culture of the bait strain was prepared by inoculating one colony into 50 ml of SD/-Trp liquid medium. The cells were incubated with shaking at 30°C until the OD_600_ reached 0.8, and then pelleted by centrifugation (1000 x g for 5 min) and resuspended in SD/-Trp medium to a cell density of 1x10^8^ cells. The Y2HGold[pGBKT7-JAK3] bait was combined with 1 ml of the library strain in 50 ml of 2x YPDA liquid medium (containing 50 µg/ml of kanamycin) and the mating mixture was incubated at 30°C for 24 hours with slow shaking at 50 rpm. After 20 hours, a drop of the culture was observed under a phase contrast microscope (40X) to check for the presence of zygotes representing two haploid parental cells containing interacting proteins. After an additional 4-hour incubation, cells were pelleted (1000 x g for 5 min), rinsed with 0.5x YPDA and resuspended in 0.5x YPDA. From this mated culture, 100 µl of 1/10, 1/100 and 1/1000 dilutions were spread onto each of the following agar plates: SD/-Trp, SD/-Leu, and SD/-Leu/-Trp (DDO) in order to calculate the number of clones screened. The remainder of the mated culture was spread onto 150 mm DDO/X/A agar plates and all plates were incubated at 30°C for 5 days. Diploid clones growing on the DDO/X/A selective media (blue colonies) were picked to identify and eliminate duplicate clones by PCR using the Matchmaker insert check PCR mix 2 (Clontech) followed by PCR product analyses on 0.8% TAE agarose/EtBr gels. These diploid clones were then spread onto higher stringency QDO/X/A agar plates. All QDO/X/A positive interactions were further analyzed to verify that the interactions were genuine. To distinguish genuine positive from false positive interactions, competent Y2HGold cells were co-transformed with pGBKT7-JAK3 bait and pGADT7-GC prey on one side and with pGBKT7 and pGADT7-cDNA prey on the other side. The two co-transformations were plated on DDO/X and QDO/X/A agar plates and incubated at 30°C for 5 days. Only library plasmids responsible for the activation of all four-reporter genes were rescued and isolated using the Easy yeast plasmid isolation kit (Clontech). To generate sufficient plasmid to allow their characterization by sequencing, prey plasmids were used to transform Stellar competent bacteria that were plated on LB agar plates with 100 µg/ml of ampicillin (Clontech). The prey inserts were identified by sequencing and nucleic acid sequences were verified for the presence of an open reading frame fused in frame to the *GAL4* AD sequence. Prey insert sequences were compared to GenBank sequences via BLAST analysis.

***Co-IP confirmation of protein interactions***

Physical interaction between JAK3 and candidate partners was confirmed by *in vitro* co-immunoprecipitation assay using the Matchmaker Co-IP system (Clontech). The universal In-Fusion primers provided in the Matchmaker Co-IP kit were used for directional PCR cloning of inserts from the pGBKT7 and pGADT7-Rec yeast two-hybrid vectors into the specified sites in the pAcGFP1-C and pProLabel-C vectors to generate in-frame AcGFP1-bait and pProLabel-prey fusions. The AcGFP BD FWD/REV primers allow amplification of bait sequences from pGBKT7 for in-frame In-Fusion PCR clon­ing into the pAcGFP1-C vector linearized with SalI/HindIII. The PL AD FWD/REV primers amplify prey sequences from pGADT7-Rec for in-frame In-Fusion PCR cloning into the pProLabel-C vector linearized with SalI/BamHI. The PCR products (5 µl) were run on a 1% agarose/TAE/EtBr gel alongside a standard to assess the yield and specificity of the products. The bait sequence PCR amplified from the pGBKT7-based vector was further digested with 1 µl of DpnI enzyme (for 60 min at 37°C) to reduce uncut vector background that would result from PCR template containing the same kanamycin resistance antibiotic marker as the cloning vector pAcGFP1-C. The JAK3 bait was then cloned into the linearized pAcGFP1-C vector and its potential prey partners from the GC-cDNA library were cloned into the linearized pProLabel-C vector using the In-fusion HD EcoDry cloning kit (Clontech). The cloning reaction mixture was used to transform Stellar competent bacteria cells (Clontech) and plasmid DNA was isolated by miniprep (Qiagen). The presence of inserts was determined by restriction digestion using SalI/HindIII and SalI/BamHI enzymes, respectively for pAcGFP1-C and pProLabel-C.

The two plasmid constructs were used to co-transfect HEK 293 cells using the CalPhos Mammalian transfection kit (Clontech) as recommended by the manufacturer. Fourthy-eight hours post-transfection, cells were collected and cell lysates were prepared for the co-IP assay. Each lysate sample was incubated with 1 µl of the anti-AcGFP polyclonal antibody on a rotator at 4°C for 2 hours. After the antibody incubation, the entire volume of each sample was transferred to a tube containing washed Protein G Plus/Protein A Agarose Beads and incubated overnight at 4**°**C with gentle rotation. Beads were thereafter gently pelleted by centrifugation at 4**°**C, 5000 x g for 10 seconds followed by washing. The samples were subjected to SDS-PAGE analysis and blotted with specific prey antibodies to confirm the presence of prey proteins physically interacting with JAK3. The selected preys were leptin receptor overlapping transcript-like 1 (LEPROTL1), inhibin beta A (INHBA), and cyclin-dependent kinase inhibitor 1B (CDKN1B). In addition, the luminescence generated as ProLabel enzymatic activity was measured in protein samples from cells co-transfected with pAcGFP1-JAK3 and pProlabel-LEPROTL1, pProLabel-INHBA or pProLabel-CDKN1B, and from the control cells transfected either with pAcGFP1-JAK3 alone (experimental control), with pAcGFP1-Lam and pProLabel-T (negative control) or with pAcGFP1-53 and pProLabel-T (positive control).
